# Design, Development and Optimization of a Functional Mammalian Cell-Free Protein Synthesis Platform

**DOI:** 10.3389/fbioe.2020.604091

**Published:** 2021-02-02

**Authors:** Chiara Heide, Gizem Buldum, Ignacio Moya-Ramirez, Oscar Ces, Cleo Kontoravdi, Karen M. Polizzi

**Affiliations:** ^1^Department of Chemical Engineering, Imperial College London, London, United Kingdom; ^2^Department of Chemistry, Imperial College London, London, United Kingdom; ^3^Imperial College Center for Synthetic Biology, Imperial College London, London, United Kingdom; ^4^Institute of Chemical Biology, Imperial College London, London, United Kingdom

**Keywords:** cell-free protein synthesis, Chinese hamster ovary cells, cell extract, synthetic biology, *in vitro* transcription-translation, coupled batch reactions

## Abstract

In this paper, we describe the stepwise development of a cell-free protein synthesis (CFPS) platform derived from cultured Chinese hamster ovary (CHO) cells. We provide a retrospective summary of the design challenges we faced, and the optimized methods developed for the cultivation of cells and the preparation of translationally active lysates. To overcome low yields, we developed procedures to supplement two accessory proteins, GADD34 and K3L, into the reaction to prevent deactivation of the translational machinery by phosphorylation. We compared different strategies for implementing these accessory proteins including two variants of the GADD34 protein to understand the potential trade-offs between yield and ease of implementation. Addition of the accessory proteins increased yield of turbo Green Fluorescent Protein (tGFP) by up to 100-fold depending on which workflow was used. Using our optimized protocols as a guideline, users can successfully develop their own functional CHO CFPS system, allowing for broader application of mammalian CFPS.

## Introduction

Cell-free protein synthesis (CFPS) is an emerging research field. Originally developed to decipher the genetic code (Nirenberg and Matthaei, 1961), CFPS has recently become a powerful tool, providing new opportunities for protein expression, metabolic engineering, therapeutic development, education, and more (Gregorio et al., 2019). CFPS has several advantages over traditional cell-based expression including an open reaction environment, which allows addition of enhancers and non-natural compounds to facilitate protein expression, lack of requirement to maintain cells in a living state, and the ability to direct all energy and cellular machinery to translate mRNA encoding the protein of interest.

Among CFPS platforms, eukaryotic systems are of increasing interest due to their ability to produce post-translationally modified proteins (Mikami et al., 2006a; Brödel et al., 2013; Zemella et al., 2018). Systems have been developed based on yeast (Hodgman and Jewett, 2013; Gan and Jewett, 2014; Aw and Polizzi, 2019; Zhang et al., 2020), plants (Madin et al., 2000; Murota et al., 2011; Buntru et al., 2014, 2015), insect (Ezure et al., 2006, 2010; Madono et al., 2011; Richter et al., 2014; Stech et al., 2014), and mammalian cells (Stavnezer and Huang, 1971; Shields and Blobel, 1978; Jackson and Hunt, 1983; Starr and Hanover, 1990; Katzen and Kudlicki, 2006; Mikami et al., 2006a,b; Mikami et al., 2008; Brödel et al., 2014; Yadavalli and Sam-Yellowe, 2015; Thoring et al., 2017; Burgenson et al., 2018; Thoring and Kubick, 2018). Mammalian systems are of particular interest due to their ability to produce glycoproteins with human-like N-linked glycosylation. This has led to the development of CFPS platforms for human (Mikami et al., 2008; Yadavalli and Sam-Yellowe, 2015; Burgenson et al., 2018) and Chinese Hamster Ovary (CHO) cells (Brödel et al., 2014, 2015; Martin et al., 2017; Thoring et al., 2017; Thoring and Kubick, 2018) among others. Among previous work demonstrating CFPS using CHO, only one group has reported the successful development of a reliable CFPS platform so far (Brödel et al., 2014, 2015; Thoring et al., 2017; Thoring and Kubick, 2018), while others have relied on the use of the commercial “1-step CHO High Yield *In vitro* Translation (IVT)” kit (Thermo Scientific, West Palm Beach, FL, now discontinued) (Martin et al., 2017). Although the cost of commercial kits is substantial, they simplify the protein expression workflow and offer standardized reagents for reproducible CFPS performance. However, since the product details of the commercial kits are not disclosed and reagents are provided as pre-mixed stocks, they have limited flexibility for customizing the reaction for different purposes.

It is therefore of great interest to increase the understanding of mammalian-based CFPS platforms and promote the development of in-house systems with improved protein yields. In this methods paper, we share our scientific insights and technical protocols with the cell-free community. Specifically, we developed a CFPS based on suspension-adapted CHO cells that includes the supplementation of two accessory proteins to increase protein yields. We studied three variations of implementing these accessory proteins and show increased expression of tGFP in all cases.

Batch reactions are well-suited to high-throughput screening as they can easily and quickly be prepared. Yields of CFPS platforms range from 1 μg/mL to 2.3 mg/mL in batch mode depending on the complexity of proteins (Kobs, 2008; Caschera and Noireaux, 2014). In CHO cell-based CFPS systems, total protein yields of 9–51.3 μg/mL have been achieved in batch mode (Brödel et al., 2014; Stech et al., 2017). Our cell-free system generates yields of up to 20 μg/mL of tGFP with accessory protein supplementation, which is in line with previously reported yields from CHO cell-based mammalian CFPS. This technical protocol should enable complete novices to successfully develop their own CHO CFPS system with results comparable to our reported data.

## Materials and Methods

### CHO Cell Culture and Lysate Generation

The CHO cell lysate was prepared from FreeStyle™ CHO-S cells (Invitrogen, UK) cultured in Erlenmeyer flasks (Corning, Netherlands) using chemically defined animal component-free Gibco™ 1X CD CHO medium (Invitrogen, UK), supplemented with 8 mM L-Glutamine (Thermofisher Scientific, UK) and 10 ml/l of 100X hypoxanthine/thymidine supplement (Thermofisher Scientific, UK). Cells were revived by thawing in a water bath at 37°C, followed by centrifugation to remove the freezing medium and resuspension in 10 mL of complete growth medium. Cultures were seeded at an initial density of 3 × 10^5^ cells/ml in 125 ml vented Erlenmeyer flasks and subcultured every 3–4 days in complete growth medium at a seeding density of 2 × 10^5^ cells/ml. Cells were subcultured at least twice before use for extract preparation. Cultures were maintained in a humidified incubator on an orbital shaking platform rotating at 125 rpm at 37°C and 5% CO_2_. Approximately 200–250 ml of CHO-S cells at a density of ~4 × 10^6^ cells/ml were required for the preparation of a few ml of lysate and culture viability of >90% were required for active lysates.

With the exception of the energy content studies, CHO cells were harvested from the batch culture at mid-log phase (day 4). Cells were collected via centrifugation for 5 min at 200 × g at 4°C and washed twice with ice-cold 25 ml HEPES-based buffer (40 mM HEPES-KOH (pH 7.5), 100 mM NaOAc, and 4 mM DTT, 1/per 50 ml complete EDTA-free Protease Inhibitor Tablet). To prevent degradation during the lysate preparation, the cell suspension was mixed gently and the pellets re-suspended by pipetting. For each wash step, the cells were centrifuged for 5 min at 200 × g at 4°C except for the final wash, when the cells were centrifuged for 10–15 min to ensure they were fully pelletized prior to final re-suspension.

Lysates were prepared in a cold room and handling time was kept to a minimum to prevent loss of activity. The pellet was re-suspended in HEPES-based buffer to a final cell density of ~4.0 × 10^8^ cells/mL, transferred into pre-cooled 3 or 5 ml glass Snap cap vials, and closed with a transparent plastic lid. Two holes were punched into the lid- one to insert the needle and a second to avoid vacuum formation. The cells were mechanically lysed by pulling and pushing the cell suspension through BD Precisionglide® 23-, 25-, 27-gauge, L 1 1/4 in. needles using sterile, disposable 2 ml BD Plastipak™ syringes. The lysis procedure was started with the largest needle size (23-gauge) and when the resistance of the cell suspension noticeably decreased, smaller needle sizes were used to increase the shear forces and disrupt the remaining cells. The progress of lysis was observed through a light microscope and was deemed complete when over 90% of cells were lysed.

The crude lysate was transferred into 1.5 ml Eppendorf tubes and centrifuged at 10,000 × g, 4°C for 10 min to remove the nuclei and cell debris. The supernatant was transferred into fresh 1.5 ml Eppendorf tubes. To degrade residual DNA, the supernatant was treated with micrococcal nuclease at room temperature. 100 mM CaCl_2_ was added at a final concentration of 1 mM, followed by 10 U/mL S7 nuclease (M0247S, NEB UK) and the lysate incubated for 2 min at room temperature. The reaction was stopped by adding EGTA to a final concentration of 6.7 mM. Finally, the lysate was flash-frozen in 200 μl aliquots using liquid nitrogen to avoid repeated freeze-thaw cycles and stored at −80°C.

### ATP and GTP Quantification

The nucleotide content was determined using high-performance ion exchange chromatography (HPAEC) as previously reported by del Val et al. (2013). In brief, CHO cells were harvested, washed and quenched using ice-cold 0.9% w/v aqueous NaCl solution. One volume of cell culture sample (1.5 × 10^7^ cells) was added to four volumes of quenching solution. The mixture was centrifuged (1,000 g, 1 min, 4°C), and the cell pellet was re-suspended in a second volume of quenching solution (ice-cold 0.9% w/v NaCl). This suspension was centrifuged again (1,000 g, 1 min) to obtain the cell pellet for acetonitrile extraction. To isolate the intracellular nucleotides, the cell pellet was then re-suspended in 3 ml of ice-cold 50% v/v aqueous acetonitrile solution. 2 μl of 20 mM GDP-Glc were added as an internal standard and the resulting suspension was incubated on ice for 10 min. The sample was centrifuged (0°C, 18,000 g, 5 min) and the supernatant was collected and dried at room temperature using a SpeedVac (Savant, USA). The dried extract was re-suspended in 240 μl of deionized water and stored at −80°C until analysis. The sample was thawed at room temperature and filtered using 0.2-μm sterile filtered Sartorius™ Minisart™ Plus Syringe Filters before analysis by HPAEC using an Alliance HPLC system (Waters) equipped with a CarboPac PA-1 column and a PA-1 guard column (Dionex, USA). HPLC-grade GTP and ATP were used to create a standard curve for the quantification of the intracellular GTP and ATP content of the cells.

### Cell-Free Protein Synthesis Reactions

The reaction mix contains a large number of reagents and its activity is highly dependent on its preparation speed and method. Due to its complex composition, preparation of the mix is highly susceptible to pipetting errors and reagent degradation. Hence, in order to obtain comparable data for assessing the effect of lysis preparation and accessory proteins, we used the commercial reaction mix. The reactions were performed using the optimized protocol of the “1-Step Human Coupled IVT Kit” (ThermoScientific, West Palm Beach, Fl) as a guideline.

All CFPS reactions were carried out in coupled batch mode in a total reaction volume of 25 μL.The reactions were composed of four different pre-prepared components (template DNA, lysate, reaction mix, and accessory proteins). For each 25 μL reaction, 1 μg template DNA was added. While a reaction mix based on previous work was initially used for the CFPS reactions (Aw and Polizzi, 2019), in the work described here we used the reaction mix supplied with the commercial kit to enable direct comparison. All components were thawed on ice and mixed together at room temperature. The lysate was incubated with the accessory proteins for 10 min and then the reaction mix and the DNA were added. The plates were sealed with BreatheEasy sealing membrane (Sigma Aldrich). CFPS was conducted overnight at 30°C in a 384-well plate (Corning) in a Synergy HT Microtiter Platereader (BioTek, Winooski, VT) with shaking on the medium setting. The model protein tGFP was expressed using the positive control (pT7CFE1-tGFP-CHis) vector included in the 1-Step Human High-Yield IVT Kit (Thermo Scientific, West Palm Beach, FL).

### Functional tGFP Quantification

tGFP protein yields were quantified by fluorescence measurement using a Synergy HT Microtiter platereader (BioTek, Winooski, VT) with an excitation wavelength of 485 nm and an emission wavelength of 528 nm. Recombinant tGFP from Evrogen (Cambridge Bioscience, UK) was used to establish a standard curve to convert fluorescence units into concentrations.

### Recombinant Expression and Purification of Accessory Proteins

Expression and purification of K3L, as well as full-length and truncated (Δ1–240) GADD34 was conducted as described in Mikami et al. using *E. coli* C41 (DE3) (Lucigen, Middleton, WI, USA) as the expression host (Mikami et al., 2006a, 2010a). The plasmids pGEX-6P-GADD34-FLAG and pTac-His-K3L were a kind gift from Hiroaki Imataka (University of Hyogo). The N-terminally truncated form of GADD34 was amplified by PCR and cloned in to pGEX-6P-1. After purification, K3L and both versions of GADD34 were subsequently buffer exchanged with 20 mM HEPES pH 7.0–7.6 (Sigma-Aldrich), and concentrated using Vivaspin 500 centrifugal concentrators with a molecular weight cut-off (MWCO) of 3 kDa and 30 kDa (Sigma-Aldrich, St. Louis, MO), respectively. For maximal activity of the accessory proteins, the purification and concentration steps were conducted in a cold room.

### Transient Expression of Truncated GADD34 in CHO Cells

The truncated GADD34 sequence (Δ1–240) was cloned into the mCherry2-C1 backbone (Addgene plasmid #54563), a kind gift from Michael Davidson, in frame with the mCherry coding sequence to generate the pmCherry-Trunc-GADD34 construct. pmCherry-Trunc-GADD34 was prepared using the Endotoxin-free Maxi kit (Qiagen, UK) following manufacturer's instruction and the purified plasmid was diluted to 1 μg/μL.

FreeStyle™ CHO-S cells (Invitrogen, UK) were used for transient expression of GADD34Δ1–240 as a C-terminal fusion with mCherry. Culturing conditions were the same as previously described. The cells were revived in a 125 mL polycarbonate sterile Erlenmeyer shake flask containing 30 mL of pre-warmed FreeStyle™ CHO Expression Medium (Thermo Scientific, UK) supplemented with 8 mM L-Glutamine (Sigma-Aldrich, UK). Seventy-two hours post-revival, the cells were subcultured at an initial seeding density of 0.3 × 10^6^ viable cells/mL in pre-warmed medium, followed by subsequent subculturing at 1 × 10^6^ viable cells/mL every 3 days. A minimum of five passages occurred before transfection to allow for cell recovery. Forty-eight hours prior to transfection, cells were passaged to obtain a density of 4–8 × 10^6^ cells/mL on transfection day. Immediately prior to transfection, the culture was diluted with complete medium to a final density of 2 × 10^6^ cells/mL in a 500 mL Erlenmeyer flask containing 80 mL culture medium.

The transfection complex consisting of 8 mL FreeStyle CHO Expression medium, 96 μL plasmid DNA, 80 μL TransIT-PRO Reagent (Cambridge Bioscience, UK), and 40 μl PRO Boost Reagent (Cambridge Bioscience, UK) was prepared. TransIT-PRO and PRO Boost Reagents were incubated at room temperature and gently vortexed prior to use. The plasmid DNA was diluted with the FreeStyle CHO medium and after gentle mixing, TransIT-PRO Reagent and PRO Boost Reagent were successively added. The prepared transfection mixture was incubated at room temperature for 10 min to allow sufficient time for complexes to form. After adding the transfection complex to the cell culture, the transfected cells were incubated for 3 days under the same culturing conditions previously described. Three days after transfection, the cells were harvested and lysate was prepared as described previously. A culture viability above 85% was required to produce active lysates. Lysates with transiently expressed mCherry-GADD34Δ1–240 were stored and handled in the same way as lysates without transient expression.

To calculate the amount of expressed mCherry- GADD34Δ1–240 fusion protein, 5 × 10^6^ cells were washed twice in cold PBS and pelleted by centrifugation at 2,500 × g for 5 min. 1 mL of RIPA buffer (Thermofisher Scientific, UK) was added to the cell pellet. The re-suspended pellet was incubated on ice with gentle shaking for 15 min. The mixture was centrifuged at ~14,000 × g for 15 min to pellet the cell debris and supernatant was used to perform fluorescence measurements. An mCherry standard curve was prepared using mCherry protein (Biovision, mCherry Quantification Kit) serially diluted in RIPA buffer with a range of 50 ng to 0.8 μg. The amount of expressed mCherry-GADD34Δ1–240 fusion in the lysate was calculated as 0.18 μg/μL.

### Statistical Analysis

Data was analyzed via one-way analysis of variance (ANOVA), followed by Student's *t*-test using a two-tailed distribution. A value of *p* < 0.05 is denoted by ^*^, *p* < 0.01 by ^**^, *p* < 0.001 by ^***^.

## Results

### Generating Functional CHO Cell Lysates

Our initial aim was to develop functional CHO cell lysates, which involved the culture and characterization of CHO cells, the determination of optimal cell harvesting time and the identification of a successful method to lyse the cells while retaining the activity of the intracellular machinery. We initially evaluated the growth characteristics of two parental CHO cell lines, CHO-S and CHO-K1. CHO-K1 was used in previous work (Stech et al., 2013), but this cell line is subject to clumping even though it is suspension adapted, making it more difficult to efficiently lyse (Supplementary Figure 1). Moreover, CHO-S cells recover more quickly from cryopreservation and have a higher growth rate, leading to a 1.5-fold higher cell density on day 5 of culture, which increases the speed of lysate preparation. Therefore, we chose CHO-S for all further work.

**Figure 1:**
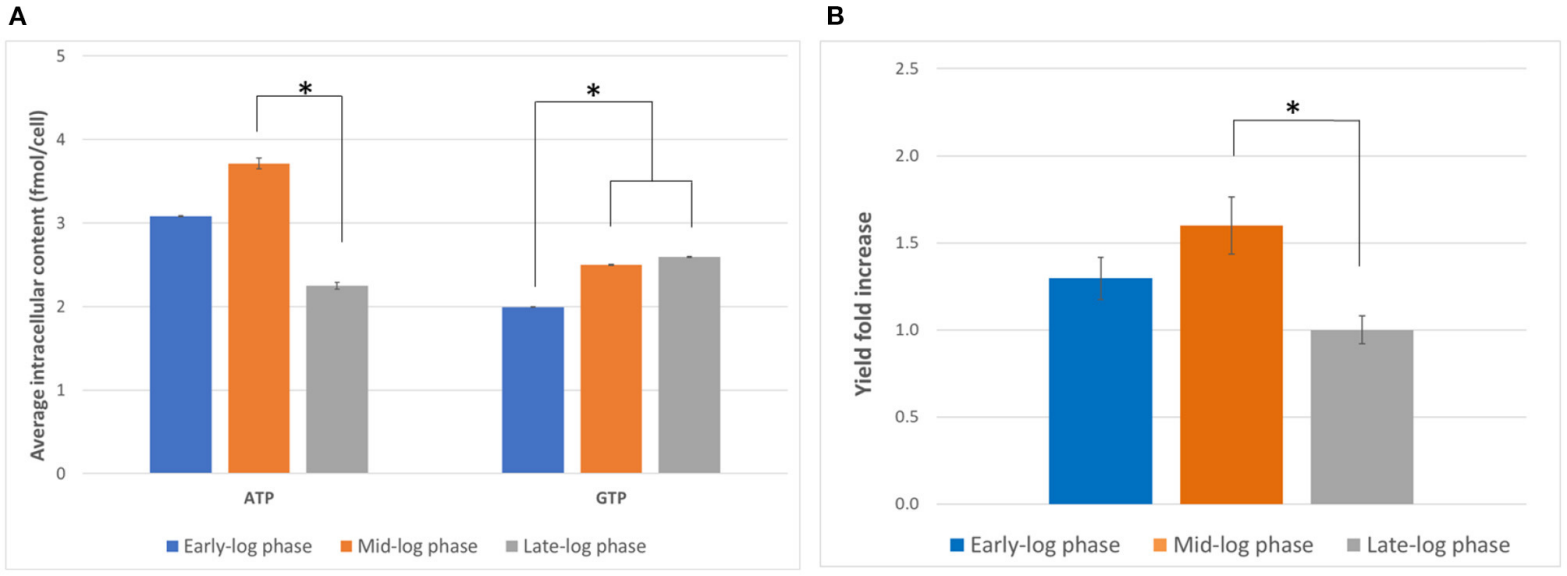
Determination of optimal harvest time. **(A)** Intracellular ATP and GTP content (in fmol/cell) on days 3, 4, and 5 (early-, mid-, and late-log phase, respectively) of culture. Error bars represent the standard deviation of the mean of three samples taken from independent culture flasks. **(B)** GFP expression using lysates harvested and prepared on the three harvesting days. The positive control plasmid, the accessory proteins, and the reaction mixtures from the 1-Step Human Coupled IVT Kit were used to facilitate comparison. Error bars represent the standard deviation of the mean of three independent CFPS reactions (*p < 0.05). Early-log phase: day 3, mid-log phase: day 4, late-log phase: day 5. Measurements were corrected for a negative control (NC, no plasmid addition).

The characterization of the cell culture is a crucial prerequisite to find the best harvesting conditions for generating active lysate. Harvesting the cell at the right time can lead to improved CFPS yields and better cost efficiency. Therefore, we first evaluated the effect of the day of harvest on the activity of the lysate generated. We hypothesized that the nucleotide triphosphate content at the harvest point might affect the translational activity by influencing the amount of available energy for the reaction as well as the phosphorylation state of translational machinery (Jewett et al., 2009). Cells were harvested on days 3, 4, and 5, corresponding to early, mid, and late exponential phase, respectively, and the ATP and GTP content were measured by anion exchange chromatography. Overall, the ATP content was more variable than the GTP content and peaked on day 4 (Figure 1A). On the other hand, while the GTP content was statistically lower on day 3 than day 4 or 5, no statistically significant difference between the GTP content on day 4 and day 5 could be observed (*p* = 0.37). Based on the results evaluated, day 4 appeared to be the optimal harvest day with respect to energy content.

We then compared the yields of the model protein tGFP from CFPS using the lysates prepared from cells harvested on days 3, 4 and 5 (Figure 1B). We observed the highest tGFP expression in reactions using lysates from cells harvested on day 4, followed by day 3 and subsequently day 5. The results indicate day 4 is the optimal harvest time for preparing lysates with maximal activity. While the reason for this observation requires further study, the ATP content might be an indicator of the overall status of the cell. For example, ATP availability might correlate with the phosphorylation state of translation initiation factors or the expression level of metabolic enzymes. This is in contrast with previous work that reported the highest activity in extracts from cells harvested from late-log phase (Brödel et al., 2015). However, different cell lines were used (CHO-S vs. CHO-K1) and the mode of culture was also different (shake flasks vs. bioreactors), suggesting that the optimal harvest time may vary depending on the cell line and culture conditions. It is hence important to characterize a chosen cell line to find the optimal harvesting time for each growth condition.

**Figure 2:**
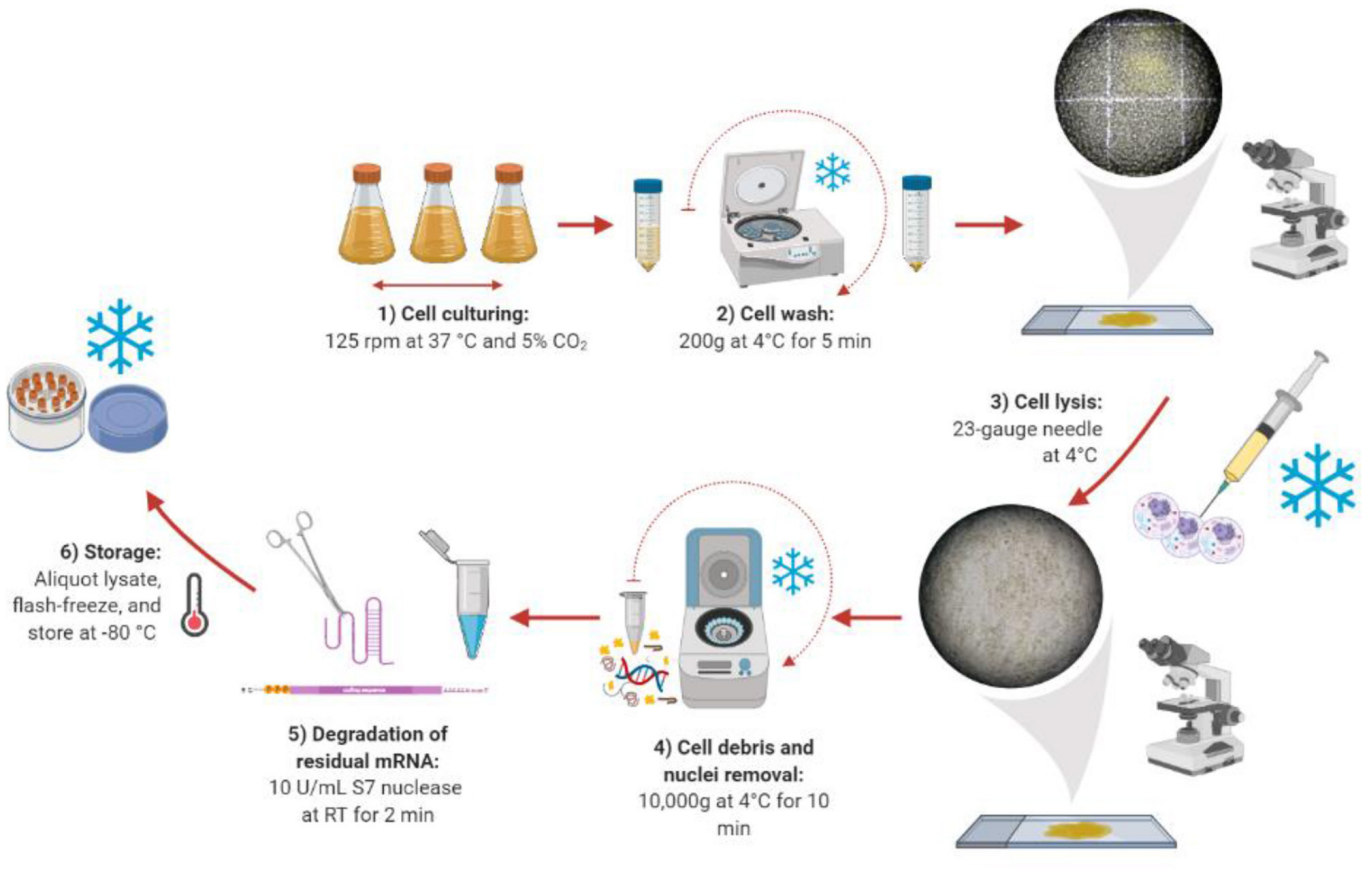
Lysate preparation workflow. Created with Biorender (biorender.com).

Once the optimal harvest day was found, we compared a range of lysis methods to identify the best method for generating active lysates. The ideal cell lysis method balances efficient disruption of the cell membrane with retaining the activity of the cellular machinery. We began by taking the protocol previously published by Brödel et al. (2014, 2015), which recommends using 20-gauge needles, as a starting point. However, the lysis efficiency was lower than expected (Supplementary Figure 2). Therefore, different lysis methods (sonication, homogenization, nitrogen cavitation, freeze-thaw cycles, and needles) were studied. Among the different approaches, only the “needle method” proved to be successful in efficiently lysing the cells (Figure 2). However, much smaller needle sizes were required (23-gauge, 25-gauge, and 27-gauge used in succession), possibly due to the smaller size of CHO-S cells compared to CHO-K1 cells. Harsher methods like sonication or homogenization, which are traditionally used for the preparation of microbial cell extract did not prove to be successful. In these cases, the cells were lysed efficiently, but the extract was not functional (see Supplementary Table 1 for a summary). We present the details of our lysate preparation workflow in Figure 2.

### Supplementing Reactions With Purified GADD34 and/or K3L Leads to an Increase in Expression Yield

Having generated active lysates, we focused on developing our own workflows for the preparation and addition of accessory proteins to increase CFPS yields. One of the bottlenecks in eukaryotic CFPS is the phosphorylation of the eukaryotic translation initiation factor 2 (eIF2), which leads to reduced translation initiation over time (Zeenko et al., 2008). To counteract this, reactions can be supplemented with two accessory proteins, GADD34 and K3L, which have been shown to increase the activity of human cell-derived CFPS systems, but had not previously been tested in CHO CFPS (Mikami et al., 2006a, 2010b; Burgenson et al., 2018). GADD34 is a regulator that recruits protein phosphatase 1, leading to dephosphorylation of eIF2α (Novoa et al., 2001), and K3L is a viral protein that acts as a pseudo-substrate, reducing phosphorylation of eIF2α (Carroll et al., 1993).

**Figure 3:**
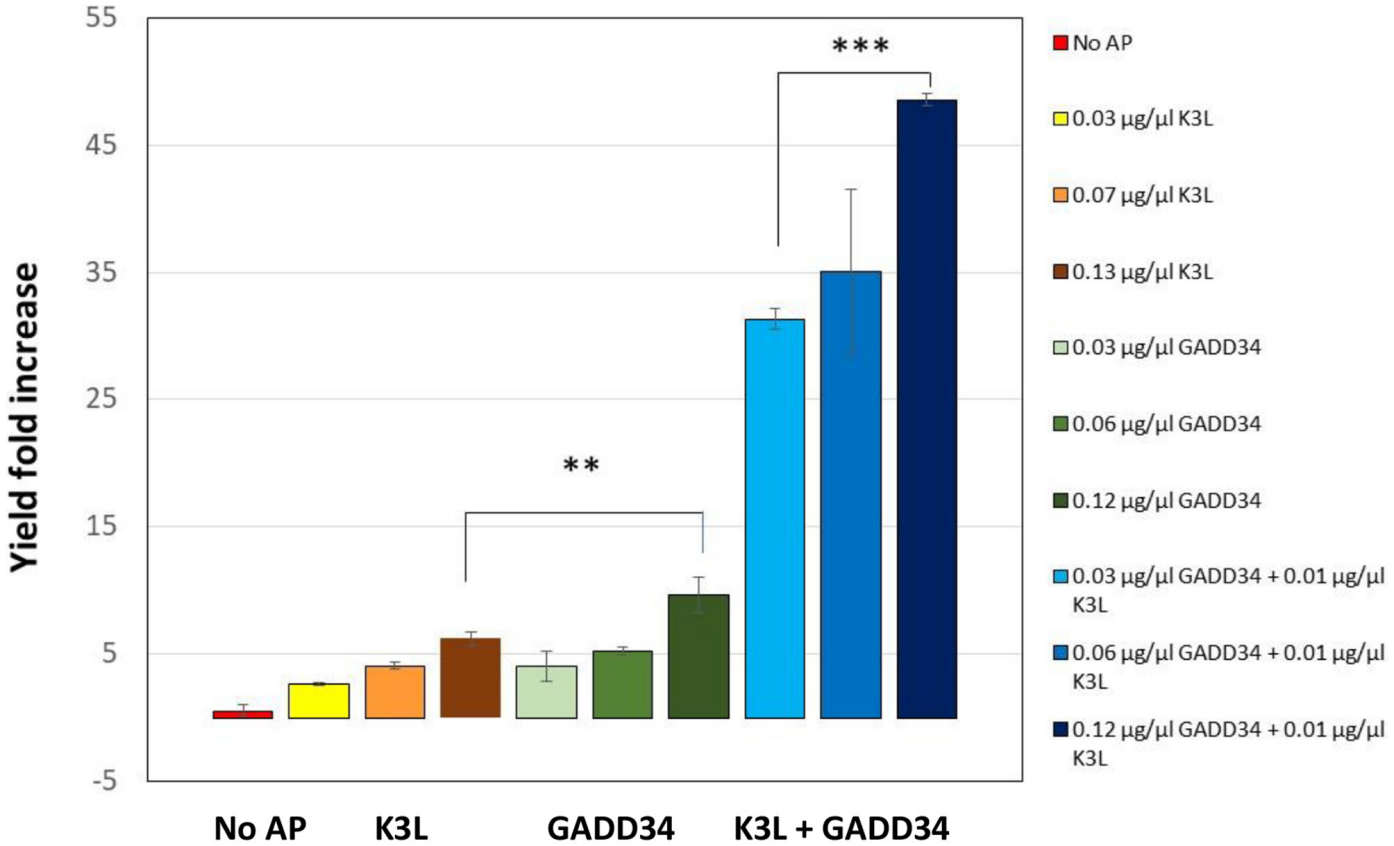
Addition of the accessory proteins GADD34 and K3L results significant increase in expression yields compared to the yield attained in their absence. GADD34 and K3L were separately and added in combination at different concentrations to the CFPS reactions. No AP refers to the CFPS reaction yield in the absence of accessory proteins, which corresponds to an average yield of ~0.4 μg/ml (range 0.05–1.08 μg/ml). Yields are normalized to individual reactions without accessory proteins run concurrently. Error bars represent the standard deviation of the mean of three independent CFPS reactions using the same lysate (**p < 0.01, ***p < 0.001).

In the first iteration, we tested the addition of different concentrations of full-length GADD34 and K3L to our system. Both proteins were recombinantly expressed in *E.coli* cells and then purified before addition (Hinnebusch, 2000). Active tGFP was produced in CFPS reactions without accessory protein addition, but yields were low and varied widely from reaction-to-reaction (average yields of ~0.4 μg/mL, range 0.05–1.08 μg/mL). Supplementing CFPS with either accessory protein alone or in combination led to an increase in tGFP yield over the non-supplemented control (Figure 3). Increasing the concentration of the accessory proteins, led to further increases in yield, presumably because of a greater degree of de-phosphorylation of the translational machinery leading to enhanced translation initiation. The concentrations tested herein were not sufficiently high to reach a plateau in tGFP expression. However, they could not be further increased due to the poor expression yields of the accessory proteins, which limited the amount of protein available for testing. There was a strong synergistic effect of adding both GADD34 and K3L that resulted in a ~50-fold increase in tGFP expression levels compared to tGFP yields attained without the addition of accessory proteins. The synergy is most likely due to the different roles that K3L and GADD34 play in reducing EIF2α inactivation. Interestingly, these results differ from those reported for HeLa extracts, where no synergy was noted (Mikami et al., 2006a). Our results further indicate that the effect of addition of GADD34 alone has a larger impact (~10-fold increase in yield) than the addition of K3L alone (~six-fold increase). This could be due to the active role of GADD34 in the recruitment of a phosphatase to dephosphorylate the translation initiation machinery, compared to the more passive role of K3L as a pseudo-substrate. With the addition of both accessory proteins, yields of tGFP approached 20 μg/mL.

### Truncation of GADD34 Improves Its Recovery Without Compromising Its Effect

Full-length GADD34 was difficult to express and purify in the quantities that were required for CFPS supplementation. However, previous work has shown that truncating membrane domain of GADD34 leads to improved expression in *E. coli* without loss of activity (Mikami et al., 2010a). Therefore, we expressed a truncated version of GADD34 (GADD34Δ1–240) and repeated the supplementation experiments. Overall, *E. coli* expressing GADD34Δ1–240 grew to a higher optical density during the expression, suggesting a reduced expression burden, with a ~1.4-fold increase in optical density (OD) at harvest (GADD34: 2.11, truncated GADD34: 2.96, Supplementary Figure 3). As a result, there was a four-fold increase in the amount of GADD34Δ1–240 obtained after purification.

**Figure 4:**
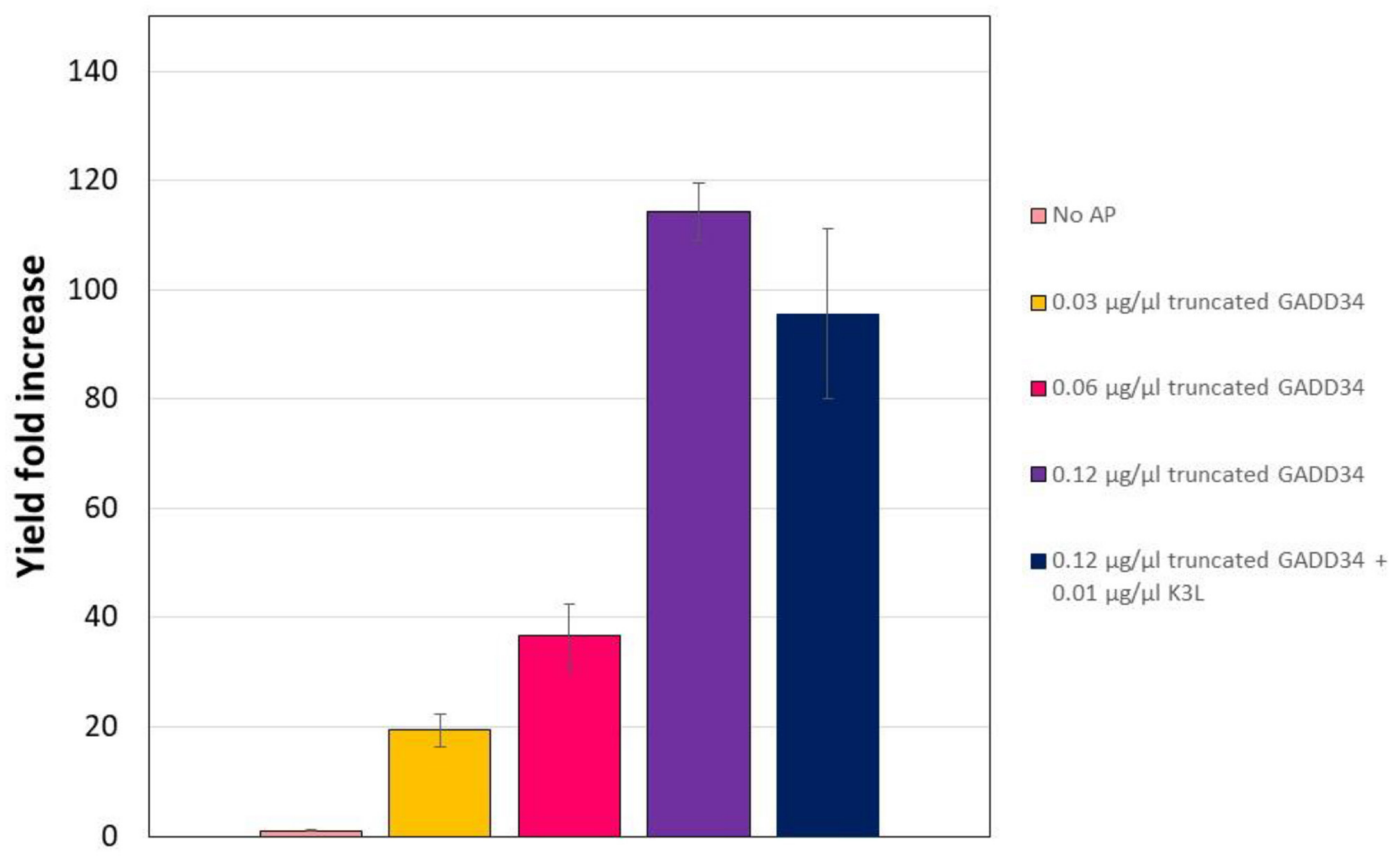
Truncation does not compromise the yield effect of GADD34 on CFPS yield. The negative control refers to the CFPS reaction yield in the absence of accessory proteins, which corresponds to an average yield of 0.13 μg/ml. Error bars represent the standard deviation of the mean of three independent CFPS reactions.

In the CHO CFPS reaction, supplementation of GADD Δ1–240 had a similar effect on protein yield as the full-length form, with increases in yield of 114-fold at the highest concentration supplemented (Figure 4). In order to assess if the truncation compromised the function, GADD Δ1–240 was tested as the same concentrations as the full-length version. However, due to variations in the control reaction without accessory protein, overall yields were lower at ~14 μg/mL. Furthermore, in contrast to the results with the full-length GADD34, there was no synergistic effect when both GADD34Δ1–240 and K3L were supplemented to the reactions.

The reason for the decreased effect of GADD Δ1–240 and K3L co-addition is not fully understood. However, it could be related to the lower purity level of the GADD34Δ1–240 protein stock produced in *E. coli* (Supplementary Figure 4). More impurities were observed for the truncated version than for the non-truncated version, which likely resulted in an overestimation of the truncated GADD34 stock concentration. It has been previously observed that the deletion of the N-terminal region improves the stability of the truncated protein, but increases the amount of co-purified bacterial proteins (Mikami et al., 2010a).

### Transient Expression of N-Terminally Truncated GADD34 in CHO-S Cells Increases CFPS Yields

**Figure 5:**
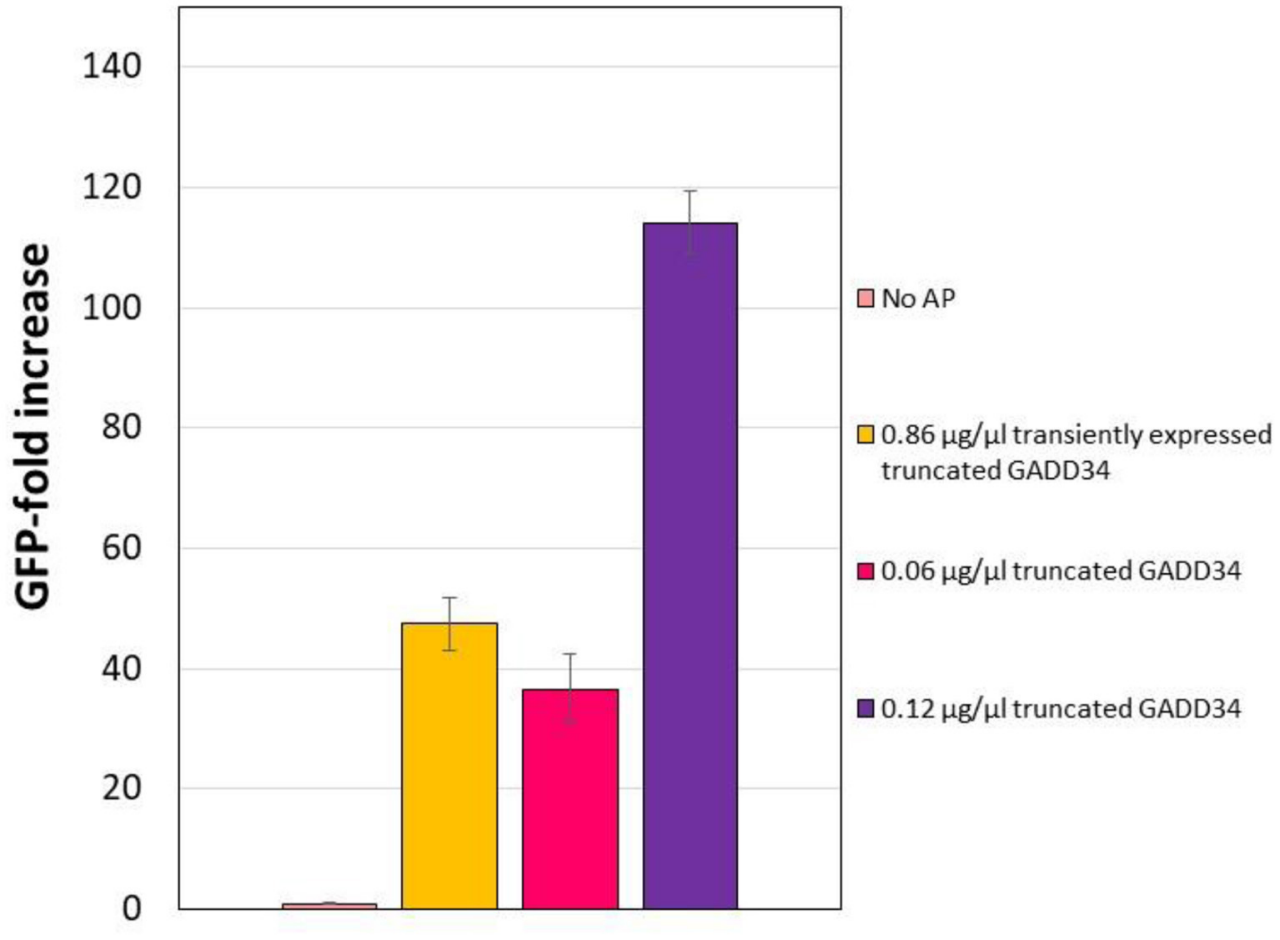
Effect of transiently expressing N-terminally truncated GADD34 in CHO cells before lysate preparation. In all cases, the negative control refers to the CFPS reaction yield in the absence of accessory proteins, which corresponds to an average yield of ~0.07 μg/ml. Error bars represent the standard deviation of the mean of three independent CFPS reactions.

Expression and purification of the accessory proteins requires additional processing steps. Therefore, we next explored the potential of transiently expressing the accessory proteins in CHO cells prior to the generation of the lysate as an alternative method that would reduce handling time and resources. GADD34 showed a greater effect on expression yields than K3L and GADD34Δ1–240 expresses at higher levels. Therefore, we focused on transient expression of GADD34Δ1–240. The GADD34Δ1–240 gene was cloned into the mCherry2-C1 plasmid in-frame with an N-terminal mCherry tag to provide a fluorescent fusion protein that was easy to detect. Cells were harvested 3 days after transient transfection and the mCherry-GADD34Δ1–240 fusion protein concentration in the lysate was estimated to be ~0.18 μg/μL (Supplementary Figure 5). Using this lysate in a CFPS reaction results in a final concentration of mCherry-GADD34Δ1–240 of 0.086 μg/μL, which is comparable to the middle concentration added in supplementation experiments (0.06 μg/μL). Overall, expressing GADD34Δ1–240 in the cells prior to the generation of the lysate resulted in a 47.5-fold increase in tGFP expression relative to the negative control in which no GADD34 was expressed (Figure 5). However, the total tGFP expression was lower in lysates made from cells transiently expressing GADD34Δ1–240 compared to those where purified GADD34 Δ1–240 was supplemented (3.5 vs. 4.8 μg/ml, respectively). We believe this is likely due to the negative effects of the transient transfection conditions on the energetic state of the cell, although we cannot rule out that the mCherry fusion leads to reduced activity of GADD34. The cell extract from the transient transfection was harvested on day 3, due to lower viability and it is well-known that the polyethyleneimine used for transient transfection reduces culture viability and may also decrease the activity level of protein machinery for CFPS. The negative effect of PEI limits the application of this approach, although stable expression of the accessory proteins in CHO extracts remains to be explored in the future. Furthermore, it may be useful to test other transient transfection methods that may have a lower impact on the physiology of the cells.

## Discussion

Mammalian CFPS are useful tools for high throughput screening and production of proteins requiring post-translational modifications. In this paper, we shared our detailed protocols for the design, development and optimization of a functional mammalian cell-free protein synthesis platform derived from cultured CHO cells. Among the required ingredients for a CFPS reaction, the cell lysate is the most labor-intensive component to prepare, requiring careful handling to protect functionality of the protein synthesis machinery. Furthermore, as the productivity of CFPS platforms depends highly on translation initiation, it is an important to enhance this step for developing robust cell-free platforms. Therefore, the focus of this study was to find the optimum method for active cell lysate preparation and to increase the protein synthesis yields via addition of accessory proteins to combat the phosphorylation of the translation machinery. Three options for increasing the expression of proteins by supplementing the reactions with accessory proteins that decrease the phosphorylation of the translational machinery were demonstrated. In general, there is a tradeoff between the effect of expression yields and the ease of implementation. Expressing the accessory proteins in a heterologous host and then supplementing them into the reaction led to higher tGFP yields overall, but is a more cumbersome protocol, which requires extra steps to purify and concentrate the accessory proteins. On the other hand, transiently expressing GADD34Δ1–240 still increases the expression of tGFP, albeit to a lesser degree due to reduced cell viability, and is much simpler. In the future, generating stable cell lines expressing the accessory proteins may provide a route for the facile generation of lysates with increased activity. By explaining our methods including all details of the developed protocols, we hope to improve the understanding of bottlenecks in CHO CFPS systems. We aim to spur greater participation in mammalian CFPS, both in the cell-free community and in bioprocessing, and hope to offer a powerful and more flexible alternative to using the available commercial kits. We believe a collaborative approach is needed to make mammalian CFPS a more widely used research tool and establish CHO CFPS as a rapid production and screening platform for therapeutic development.

## Data Availability Statement

The original contributions presented in the study are included in the article/Supplementary Materials, further inquiries can be directed to the corresponding author/s.

## Author Contributions

CH: conceptualization, methodology, investigation, data curation, writing- original draft preparation, visualization. GB: investigation, writing–reviewing and editing. IM-R: investigation, data curation, writing–reviewing and editing. OC: writing–reviewing and editing. CK and KP: conceptualization, supervision, writing–reviewing and editing. All authors read and approved the final manuscript.

## Conflict of Interest

The authors declare that the research was conducted in the absence of any commercial or financial relationships that could be construed as a potential conflict of interest.
