# Supplementary material for: Design, Development and Optimization of a Functional Mammalian Cell-Free Protein Synthesis Platform
Source: Front Bioeng Biotechnol. 2021 Feb 2;8:604091. doi: 10.3389/fbioe.2020.604091 (PMC7884609; doi:10.3389/fbioe.2020.604091)
Supplement: Supplementary file 1 [file Table_1.docx]

**SUPPLEMENTARY MATERIAL**

**Design, development and optimisation of a functional mammalian cell-free protein synthesis platform**

Chiara Heide^1,2,3^, Gizem Buldum^1^, Ignacio Moya-Ramirez^1,3^, Oscar Ces^2,4^, Cleo Kontoravdi^1*^, Karen M. Polizzi^1, 3*^

^1^Department of Chemical Engineering, ^2^Department of Chemistry, ^3^Imperial College Centre for Synthetic Biology, ^4^Institute of Chemical Biology, Imperial College London, SW7 2AZ, UK

*To whom correspondence should be addressed: cleo.kontoravdi@imperial.ac.uk, k.polizzi@imperial.ac.uk


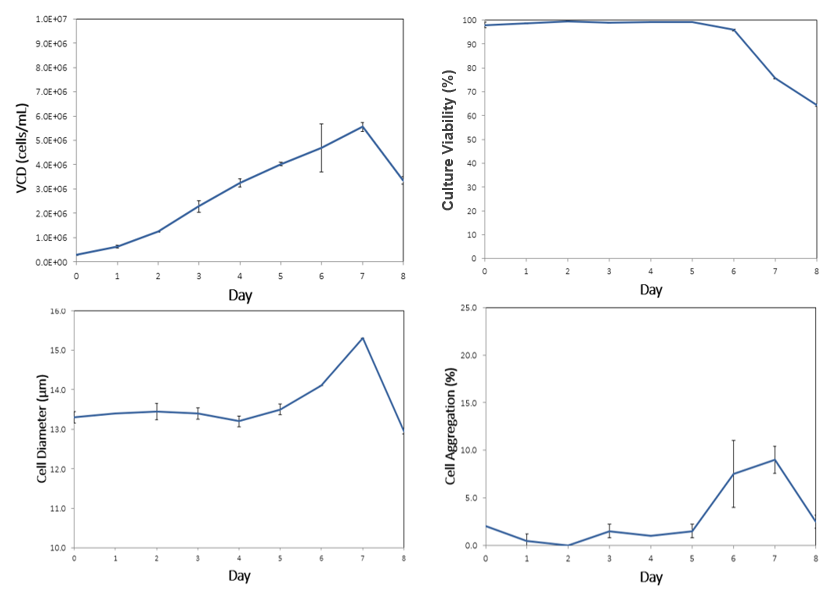


**Supplementary Figure 1**: Fed-batch cultivation and characterization of CHO-K1 cells. Cells were characterized by their viable cell density, viability, cell diameter and cell aggregation. Error bars represent the standard deviation of the mean of three samples taken from independent culture flasks.


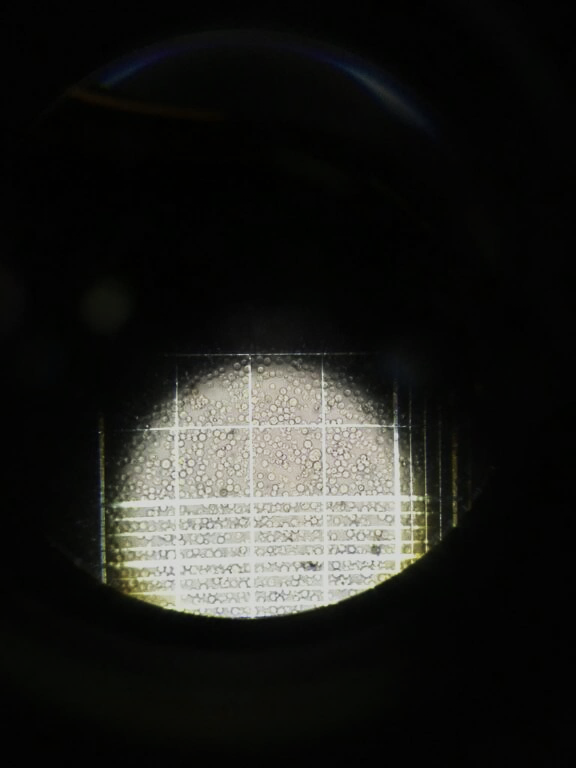


**Supplementary Figure 2**: Intact cells after lysate preparation using 20-gauge needle (1 1/2 in., Z118052). The needle was too large to effectively lyse the cells. No translationally active machinery could be released to produce active CHO lysate.


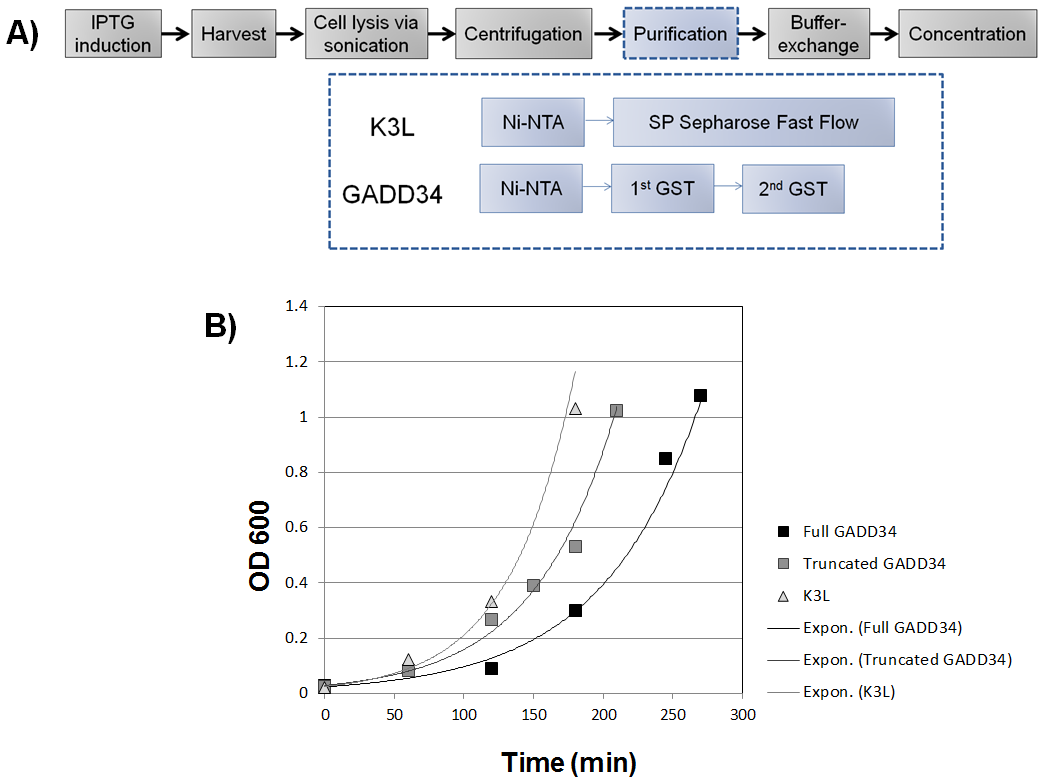


**Supplementary Figure 3:** Expression and purification of recombinant GADD34 and K3L proteins. A) Overview of the workflow for purifying the accessory proteins. GADD34 stands for the truncated version. The full-length version was purified with GST only. B) Growth curve of E. coli cells prior to IPTG induction. The truncation of GADD34 reduces the growth inhibition on the bacterial expression cells and also leads to a higher OD at harvest (GADD34: 2.11, truncated GADD34: 2.96, K3L: 3.46 after 16 hrs of expression at 20 °C using 0.1 mM IPTG in 400 ml expression volume).


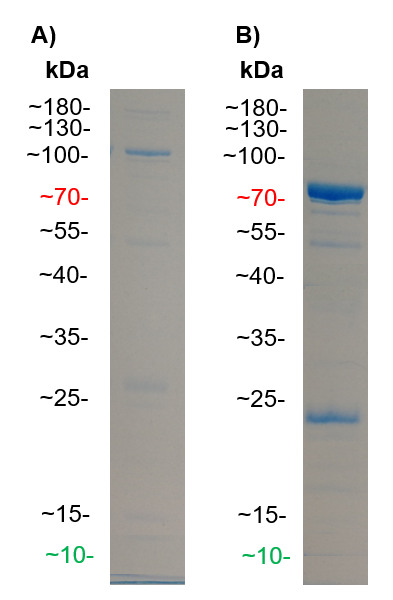


**Supplementary Figure 4:** SDS-PAGE gels of the purified stocks of A) GADD34 and B) truncated GADD34, showing the decrease in purity level for the truncated GADD34 stock.


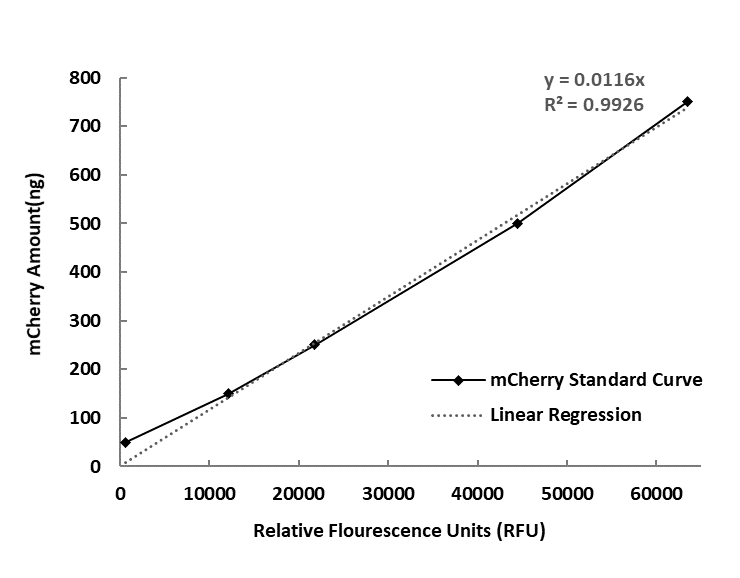


**Supplementary Figure 6:** mCherry standard curve for estimating the expression of truncated GADD34 in transient transfection. Concentrations in the range of 50 ng to 0.8 μg were used. The calculated mCherry-Truncated GADD34 fusion was 0.18 µg/µl by following equation: A/V x D (ng/μl) where: A = mCherry amount from the standard curve (632.5 ng) V = sample volume added into the reaction well (200 μl) D = dilution factor (58). Excitation wavelength: 584 nm; emission wavelength: 610 nm.

**Supplementary Table 1**: Summary of lysis efficiency

|  | **Lysis** | **Active** |
| --- | --- | --- |
| **Cell Lines** | | |
| CHO-S |  |  |
| CHO-K1 | partially |  |
| **Lysis Methods** | |  |
| Sonication |  | x |
| Needle (20 gauge) | x | x |
| Needle (23 gauge, 25 gauge, 27 gauge) |  |  |
| FastPrep Homogenization |  | x |
| Freeze-thaw cycles | x | x |
